# Large scale brain functional networks underlying non-suicidal self-injury behavior, addiction and functions in youth psychiatric disorders

**DOI:** 10.1192/j.eurpsy.2026.10254

**Published:** 2026-07-31

**Authors:** X. Liu, Y. Yang, J. Chen, Y. Zhang, D. Wang, C. Lu, F. Xu, J. Gao, Y. Yao, B. Hommel, X. Zhu, K. Wang, W. Zhang

**Affiliations:** 1 School of Psychology; 2 Shandong Provincial Key Laboratory of Brain Science and Mental Health, Shandong Normal University; 3 Shandong Mental Health Center; 4 Radiology Department of Qilu Hospital, Shandong University; 5 Medical Science and Technology Innovation Center, Shandong First Medical University & Shandong Academy of Medical Sciences, Jinan, China

## Abstract

**Introduction:**

Non-suicidal self-injury (NSSI) is a common high-risk behavior in adolescents and it occurs in various psychiatric disorders, especially major depressive disorder (MDD). It remains largely unknown whether and which brain functional networks contribute to NSSI across youth psychiatric disorders.

**Objectives:**

This study aimed to identify common brain functional networks associated with NSSI across youth psychiatric disorders, and to examine their relationships with NSSI behavior, addiction, and its functions. Furthermore, we sought to validate the generalizability of these neural correlates in independent clinical cohorts.

**Methods:**

This study analyzed functional brain imaging data acquired from 156 adolescents (MDD+NSSI group, n = 44, age = 15.32 ± 1.51; MDD-NSSI group, n = 32, age = 15.36 ± 1.96; healthy controls, n = 80, age = 15.92 ± 2.72). NSSI behavior, addiction and its four NSSI functions (internal and external emotion regulation, social influence and sensation seeking) were assessed using the Ottawa Self-injury Inventory. Using support vector machine recursive feature elimination classification and regression models, we investigated the brain functional networks that predicted NSSI. External validations were performed in an ADHD cohort (n = 40) and a transdiagnostic cohort (n = 40).

**Results:**

The brain networks related to NSSI behavior were mainly composed of inter-network connections between the fronto-parietal, motor, limbic, basal ganglia networks. These networks were also associated with NSSI addiction and its four functions. Notably, the fronto-parietal network was involved in all NSSI components. External validations in both the ADHD and the transdiagnostic cohorts validated the associations of these functional networks with NSSI severity.

**Conclusions:**

Our results demonstrate roles of the fronto-parietal, motor, limbic and basal ganglia networks in NSSI across youth psychiatric disorders, which may serve as neural markers and potential targets for prevention and intervention.

**Disclosure of Interest:**

None Declared

